# Human Cytomegalovirus and Autoimmune Disease

**DOI:** 10.1155/2014/472978

**Published:** 2014-04-29

**Authors:** Anne Halenius, Hartmut Hengel

**Affiliations:** Institute of Virology, University Medical Center, Albert-Ludwigs-University Freiburg, Hermann-Herder-Straße 11, 79104 Freiburg, Germany

## Abstract

Human cytomegalovirus (HCMV) represents a prototypic pathogenic member of the **β**-subgroup of the herpesvirus family. A range of HCMV features like its lytic replication in multiple tissues, the lifelong persistence through periods of latency and intermitting reactivation, the extraordinary large proteome, and extensive manipulation of adaptive and innate immunity make HCMV a high profile candidate for involvement in autoimmune disorders. We surveyed the available literature for reports on HCMV association with onset or exacerbation of autoimmune disease. A causative linkage between HCMV and systemic lupus erythematosus (SLE), systemic sclerosis (SSc), diabetes mellitus type 1, and rheumatoid arthritis (RA) is suggested by the literature. However, a clear association of HCMV seroprevalence and disease could not be established, leaving the question open whether HCMV could play a coresponsible role for onset of disease. For convincing conclusions population-based prospective studies must be performed in the future. Specific immunopathogenic mechanisms by which HCMV could contribute to the course of autoimmune disease have been suggested, for example, molecular mimicry by UL94 in SSc and UL83/pp65 in SLE patients, as well as aggravation of joint inflammation by induction and expansion of CD4+/CD28− T-cells in RA patients. Further studies are needed to validate these findings and to lay the grounds for targeted therapeutic intervention.

## 1. Introduction

Autoimmune disease (AID) is a complex dysregulation of immunity, resulting in loss of self-tolerance and subsequent assault on endogenous tissue or cells. Onset of autoimmunity depends both on genetic and environmental factors (e.g., viruses) and is typically driven by antibodies or T-cells reacting against self-epitopes. Self-reactive immune responses can occur only transiently and may not necessarily cause overt disease, but in many cases self-destruction of initially healthy tissue gets permanently out of control, resulting in self-perpetuating clinical disease progressing in the absence of the triggering event.

Infection with Epstein-Barr virus (EBV), a human pathogenic *γ*-herpes virus, has been linked with the risk to develop multiple sclerosis (MS), an autoimmune disorder of the central nervous system characterized by the formation of lesions, inflammation, and the destruction of myelin sheaths of neurons [1]. A similar matter of recent debate is if and how infection with human cytomegalovirus (HCMV), another human herpes virus, could be linked with certain autoimmune diseases and how both conditions could interfere with each other. Several features including its extensive manipulation of adaptive and innate immune functions [2–5], the very large coding capacity [6], its lytic replication in multiple tissues [7] both locally and systemically, its lifelong persistence during subsequent phases of latency and reactivation, and its ubiquitous prevalence in human populations readily explain why HCMV was frequently linked with AID but also with further acquired disorders like arteriosclerosis and vascular disease [8], immune aging [9], and certain types of tumors [10].

Hypothetically, the mutual influence of HCMV infection and autoimmunity could have different consequences. HCMV infection could cause, promote, or prevent AID, while conversely AID could inhibit or support HCMV reactivation from latency and/or replication. Given the fact that productive infection of HCMV usually results in cell death and thus impedes tissue integrity, HCMV replication may enhance tissue damage caused by autoimmune pathologies. Conversely, anti-inflammatory activities known to be associated with HCMV infection could also exert protective effects on the course of autoimmune diseases. On the other hand, inflammation of tissues due to AID may generate an environment preventing the reactivation and growth of HCMV, or, depending on the prevailing factors, exert proviral effects and support productive infection.

While mutual influences of HCMV infection and established AID are consequently unavoidable, any causal relationship is much harder to substantiate. A frequently discussed phenomenon in the case of turning the immune system against self-epitopes is “molecular mimicry” [11, 12]. According to this concept pathogenic foreign epitopes are highly similar to host determinants and, therefore, after activation of the immune system result in self-attack. So far, a handful of pathogenic epitopes have conclusively been shown to trigger autoimmunity by molecular mimicry [13]. However, in many cases where viruses are found to coincide with the onset of autoimmune disease, such an epitope has not been identified. Apart from that, during infection the immune system will be strongly activated: cells destroyed by viruses or by immune attack can further activate dendritic cells and macrophages. Infected tissue and activated antigen presenting cells (APC) will attract immune cells producing high levels of cytokines and chemokines, which facilitate a so-called “bystander activation” and lower the threshold for a loss of tolerance.

This review attends to give an overview of the role of HCMV for initiation and/or exacerbation of autoimmune responses. The search for published data was performed using the PubMed database and entering the keywords “cytomegalovirus” and “autoimmunity.” The autoimmune diseases, which were found most often in combination with both keywords, were then searched again in combination with cytomegalovirus alone. These diseases were systemic lupus erythematosus (SLE), systemic sclerosis (SSc), type 1 diabetes (T1D), multiple sclerosis (MS), and rheumatoid arthritis (RA).

## 2. HCMV Pathogenesis and Modulation of the Immune System by HCMV

HCMV represents a human pathogenic herpes virus belonging to the subfamily of* Betaherpesvirinae*. As all herpes viruses HCMV have a large double-stranded DNA genome and possess a formidable coding capacity giving rise to more than 750 translational products [6] and a multitude of virus-encoded miRNAs in infected cells [14, 15], reflecting the exceptional power and ability of this virus to manipulate and cope with the host. During coevolution over millions of years HCMV adapted closely to the human host and today infects 40–99% of the adult populations depending on ethnic and socioeconomic conditions. Infection in immunocompetent individuals normally proceeds unperceived. After primary infection of diverse cell types (e.g., epithelial cells of the liver, lungs, kidney, salivary glands, large intestine, placenta endothelial cells, smooth muscle cells, fibroblasts, neuronal cells, and various myeloid cells [7]), HCMV remains latent in CD34+ myeloid progenitors, from which reactivation and recurrent replication can emerge. Even though a healthy immune system controls HCMV replication, the infection can neither be eliminated by immune functions nor by antiviral drugs, precluding a state of sterile immunity. Upon failure or reduced efficiency of specific immune functions, opportunistic HCMV infections may lead to severe or even fatal illness. Common clinical findings in immunocompromised patients are fatigue, hepatitis, enterocolitis, encephalitis, pneumonitis, bone marrow failure and in AIDS patients also retinitis [16]. Moreover, HCMV infection is associated with a diminished graft survival in organ transplant recipients. Congenital HCMV infections are a leading cause of sensorineural hearing loss and permanent disability in infants [17].

Immune control of primary and latent HCMV infection is organized in a hierarchical but also redundant manner [18–20], with prominent roles for type I and type II interferons, NK cells, and CD8+ but also CD4+ T-cells, while antiviral antibodies are essential to restrict dissemination of recurrent virus. Even if a healthy immune system successfully controls infection, HCMV leads to permanent changes in the composition of immune cell populations. Steadily expanding CD8+ T-cells specific for a few HCMV epitopes dominating the memory CD8+ T-cell population is a hallmark of CMV infection and not observed for other viruses [21]. In the elderly HCMV-specific CD8+ T-cells may cover more than 20% of circulating CD8+ cells [22], a phenomenon called “memory inflation.” In addition, a higher frequency of HCMV specific CD4+ T-cells can be observed in HCMV positive persons. These cells exhibit a CD4+/CD28− phenotype and are classified as terminally differentiated effector memory cells [23]. Recently, a role for HCMV in early aging of the immune system or “immune risk phenotype” (IRP), measured as inverted CD4 : CD8 ratios, was linking HCMV to immunosenescence [24] and impaired responsiveness to vaccination [9], with possible effects seen already in young adults [25]. Moreover, induction of IL-6 and TNF*α* has been described in HCMV-positive persons [9].

Furthermore, HCMV infection results in expansion of a NK cell subset expressing activatory CD94/NKG2C receptors* in vivo* and* in vitro* [26, 27]. Independently, HCMV induces the expansion and differentiation of killer cell immunoglobulin like receptor (KIR-)expressing NK cells, manifesting as stable imprints in the NK cell repertoire. Obviously, HCMV-induced education by inhibitory KIRs is promoting a clonal-like expansion of NK cells, causing a bias for self-specific inhibitory KIRs [28].* In vitro* analysis revealed also a HCMV-driven growth potential of additional NK cell subpopulations characterized by inhibitory KIR2DL1 and KIR2DL3 as well as activatory KIR3DS1 receptors [29].

Once infected, cells become tightly controlled by HCMV, expressing numerous regulators of the cell cycle, apoptosis, cell signaling pathways, antigen presentation, and so forth (see Table 1). To date, the molecular understanding of HCMV interference with T cell responses, NK cell activation and effector function, IFN-induction, and IFN receptor signaling as well as pathways of apoptosis is most advanced but is still representing only the tip of the iceberg.

The expression profile of infected cells becomes massively modified by HCMV due to (i) transcription of HCMV genes, (ii) extensive manipulation of the cellular transcriptome due to HCMV encoded transcription factors, and (iii) the expression of HCMV miRNAs which affect both HCMV and host transcription pattern. Thus HCMV infection must lead to extensive qualitative and quantitative changes of peptide ligands presented by MHC I and MHC II molecules which are not restricted to the emergence of viral epitopes but could include also novel endogenous self-epitopes on the cell surface. Given the massive interference of HCMV with host cell gene expression on the one hand and pathways of antigen presentation on the other hand the comprehensive analysis of MHC ligandomes is a paramount goal for future studies assessing potential pathogenic mechanisms that involve molecular mimicry between HCMV and individual self-peptides.

## 3. Prevalence of HCMV IgG in Patients with Autoimmune Disease

If HCMV plays any causative role for the pathogenesis and onset of autoimmunity, it should be expected that a higher prevalence of HCMV IgG antibodies is found in patients suffering from defined types of autoimmune diseases. To gain an overview of the frequency of HCMV infection in patients with autoimmune pathologies, published data were collected and HCMV seroprevalences in defined groups of patients in comparison to reported control groups were compiled (Table 2). In some of the listed reports HCMV was not the objective of the study but used as a control parameter for EBV seroprevalence. Most of the studies have been performed in patients suffering from SLE and MS, but also studies on SSc, T1D, RA, and Sjögren's syndrome (SS) have been reported. Taking all available studies into account it is conspicuous that no clear association of HCMV infection with a specific disease can be claimed. One difficulty in establishing a connection between AID and infection is the fact that HCMV is widespread in the human population while specific autoimmune diseases are rather rare, requiring large patient cohorts to provide sufficient statistical power. Since HCMV prevalence critically depends on factors like ethnicity, age, socioeconomic conditions, and sexual lifestyle it is of high importance to analyze appropriate control group matching the patient cohort for all confounding factors. In many studies effort was given to control for age and ethnicity; however, in most cases the socioeconomic background was not accounted for. Moreover, the order of events (AID followed by HCMV infection versus HCMV infection followed by AID) was not distinguished in these studies.

### 3.1. SSc

The statistically highly significant association of HCMV infection in Swiss SSc patients (59% seropositivity in SSc patients compared with 12–21% in controls) [83] has not been observed in other studies so far [81, 82], even though higher HCMV antibody concentrations have been found in SSc patients [103, 104]. It should be mentioned that in SSc patients heterozygotes for *f* and *z* alleles of the Ig heavy chain an association with HCMV-specific antibodies was found, giving a hint for an important role of the genetic background [82].

### 3.2. SLE

Studies that found an association between HCMV and SLE disease were often performed in European countries [89–91]. Several other studies did not observe a direct association between HCMV seroprevalence and SLE [86–88]. In one of these studies HCMV seropositivity correlated significantly with Raynaud's phenomenon [90]. Further, another study reported on significantly more frequent HCMV specific IgM in SLE patients than in controls, but no difference in HCMV IgG prevalence was observed [85]. This finding could be an indication for more frequent HCMV reactivation events in SLE patients, which may occur as a result of immunosuppressive treatment. Also studies outside of Europe found higher frequencies of HCMV infection in SLE patients [81, 92] or higher HCMV IgG titers [105]. Moreover, in SLE patients with higher HCMV specific IgG titers more frequent autoantibodies could be detected [106]. It is peculiar however and not easily explainable that in that particular study a patient group positive for anti-HCMV IgM (and IgG) showed lower levels of autoantibodies against U1RNP/Sm and U1-70 k in comparison to the HCMV IgM(−)/IgG(+) group, suggesting a role for HCMV reactivation in regulation of autoantibodies.

Altogether, these findings are compatible with the notion that genetic factors in combination with HCMV infection play an important role for SLE disease onset.

### 3.3. T1D

In two independent Finnish studies no association between HCMV and onset of T1D in young children could be established [93, 94]. This result confirms a Swedish study determining the prevalence of T1D after congenital HCMV infection [107]. A single case report has been published, in which a congenitally HCMV infected child developed T1D already at the age of 13 months [108]. Thus this might represent an isolated case that occurred in combination with other unknown factors. The reported frequent finding of HCMV genomes in PBMNCs of Canadian T1D patients [109] is therefore not supported by the Scandinavian studies mentioned above, suggesting that HCMV could display a risk only for a subpopulation of affected children, rather than playing a major role for the etiology of T1D.

### 3.4. MS

In several studies HCMV has been found to negatively associate with MS [95, 97, 110]. Only one out of six studies listed here noted a higher HCMV prevalence in MS patients [96]. Whether this is a specific property of the Iranian population or if other factors might have made an impact on this study is not clear. It is interesting though that in mice a protective role for mouse CMV (MCMV) on Theiler's murine encephalitis virus induced murine model of MS has been described. Improved motor performance and a significant reduction of brain infiltrating CD3+ cells were described as a result of MCMV infection [111].

### 3.5. RA

Only few studies were found that have determined HCMV seroprevalence in RA patients: whereas higher HCMV coincidence with RA was found in one report [91], the two others did not observe this [83, 100]. All studies were carried out in Europe.

### 3.6. SS

Using only a low number of subjects no significant correlation between HCMV seropositivity and Sjögren's syndrome could be established [102], whereas in another study with small numbers of study participants higher HCMV antibody titers were reported [112].

### 3.7. Summary

According to the studies evaluated here, there is no evidence that HCMV plays a role for the onset of T1D and MS. However, concerning SLE and SSc it cannot be ruled out that HCMV indeed may play an active role in the induction of disease depending on largely unknown genetic factors. A number of case reports underline this possibility (SSc: [113]; SLE: [114–116]). For a rejection or confirmation of the HCMV hypothesis in the induction of SSC and SLE and a better clarification of HCMV-associated risk factors population-based prospective studies must be performed in the future.

## 4. Relevance of HCMV Molecular Mimicry and Bystander Effects in Autoimmune Disease

Crossreactive TCRs or immunoglobulins expressed by T- or B-cells, respectively, that have been primed against a pathogenic structure might recognize self-epitopes due to their sequence or structural similarity. This is the basis for molecular mimicry, which has been proposed to underlie aetiologically some autoimmune diseases initiated by pathogens [117]. It has been suggested that HSV-1 encoded UL6 (residues 299–314) induces autoreactive T-cells causing herpetic stromal keratitis [118]. It is a matter of debate, though, whether molecular mimicry is the key to disease development or if crossreactivity in combination with bystander functions and tissue damage is necessary to set off pathologic immune responses [119–121].

The DNA genome of HCMV encompasses about 230 kbp and was initially estimated to encode for around 200 proteins [122]. By ribosome profiling this number was recently readjusted to more than 750 encoded translational products [6]. In the context of productive infection, this massive number of proteins confronts the host with plenty of antigens, against which the immune system is simultaneously primed or boosted, thereby raising the likelihood of priming immune cells against a crossreactive epitope and taking the risk of bystander activation. So far, however, no HCMV encoded antigen has been clearly linked to an autoimmune disease.

### 4.1. HCMV and SSc

SSc is a connective tissue disorder with abnormal fibroblast cell proliferation. Endothelial cell activation leads to vascular pathology and over time to neointima formation in small and medium sized arteries. Typically, there is an increase in CD4+ and decrease in CD8+ T-cells numbers. Self-reactive antibodies against determinants of endothelial cells and topoisomerase have been shown to correlate with disease activity [123].

Interesting observations have been made concerning antibodies against the HCMV encoded protein UL94. Using a random peptide library, a peptide sequence could be identified, against which 84 out of 90 SSc patients displayed antibodies. The purified antibody bound to the cellular integrin-NAG-2 complex and UL94 (possibly also hnRNP proteins and cytochrome C) [124]. However, when reactivity against the UL94-derived peptide was determined, only 55 out of 90 patients were found to have antibodies against the peptide, raising the question whether the SSc specific antibodies were crossreactive with UL94 rather than vice versa. Information about the HCMV serostatus of the patients was not provided.

The SSc antibody was found to bind to NAG-2 both on endothelial cells and fibroblast, which resulted in major changes of the expression pattern that could be linked to previous observations in SSc patients [125]. Antibody binding to NAG-2 on endothelial cells leads to apoptosis, whereas apoptosis was not induced in fibroblasts [124, 125].

Further, the level of UL94-reactive antibodies was analysed in patients with diffuse (more prominent vascular disease) and limited forms of SSc [126]. Patients with diffuse SSc had significantly higher levels of the UL94-reactive antibody, pointing at a possible role for HCMV in disease aggravation. However, again, the HCMV serostatus of these patients was not reported. In addition to the possibility that a* de novo* antibody response against UL94 upon HCMV infection is the reason for these antibodies to arise, it is also possible that the patients might have developed these antibodies independently of HCMV, or the patients were prone to develop the antibodies and HCMV lowered the threshold and caused loss of tolerance. In the latter case, it could be possible that also another pathogen might induce such an event. To corroborate this sequence of events, further studies are needed. It should be established whether the UL94-reactive antibody is actually a true anti-UL94 antibody and, hence, associated with a positive HCMV serostatus. Are these antibodies also found in healthy HCMV positive persons and, furthermore, do HCMV negative SSc patients have less or no activity against NAG-2 and UL94?

Analysis of peptides recognized by antitopoisomerase I antibodies revealed a homologous peptide sequence present in the UL70-encoded polypeptide [127]. Experimental data that this peptide is indeed recognized by such autoantibodies is not available so far.

Evidence for association of HCMV with SSc disease is still diffuse. However, Pandey and LeRoy hypothesized HCMV to be an amplifier of SSc in a review of possible mechanisms by which HCMV could contribute to autoimmune vasculopathies [128]. Interestingly, mice with a deficiency in IFN-*γ* signaling (IFN-*γ*R KO) and increased susceptibility to MCMV showed signs of neointima formation [129]. If SSc-like disease manifestations could be found in this situation, this model could provide insight into the pathophysiology of HCMV infection eventually leading to SSc disease.

### 4.2. HCMV and SLE

Characteristic for SLE is apoptosis of endothelial cells and following atherosclerotic plaque development. Autoantibodies directed against nuclear structures (e.g., antinuclear antibodies, ANA and anti-dsDNA) are detectable years before onset of disease [130] and their levels correlate with disease severity. It is not clear though to what extent DNA antibodies contribute to the disease but immune complexes are found in temporal and spatial association with glomerular inflammation. Notably, antinuclear and anti-dsDNA antibodies were found in patients suspected to have an onset of SLE as a consequence of HCMV infection [131].

The* UL83*-encoded pp65 matrix protein has been linked to autoantibodies in SLE patients. Elevated levels of anti-pp65 antibodies were found in SLE patients compared to controls and CTD (connective tissue disease) as well as RA patients. However, in SLE patients also elevated levels of another tegument protein, pp150, were found [132], raising the possibility that anti-CMV antibodies were generally induced in these patients. In this particular publication it was also reported that an* UL83*-encoding plasmid used for immunization of NZB/W F1 mice caused production of anti-dsDNA and antinuclear antibodies, leading to more severe signs of glomerulonephritis than control plasmid immunization [132].

In a second study it was found that ca 75% of pp65 reactive antibodies from controls (healthy, RA, SS, and SSc patients) recognized the N-terminus, whereas antibodies from SLE patients were predominantly (70%) reactive against the C-terminus (amino acids 336–379) of pp65 [133]. BALB/c mice were immunized with peptides corresponding to the amino acids 1–167 or 336–439 of pp65 together with the complement component C3d as an adjuvant and the crossreactivity of the raised antibodies was analyzed. Antinuclear and anti-dsDNA antibody activity could be measured and mice immunized with the C-terminal peptide showed signs of IgG deposition on glomeruli. The C-terminal peptide resulted in antibodies reactive against HeLa cell lysates (as a measure of crossreactivity); it should be noted though that the reactivity against lysates from HCMV infected cells was also higher for these antibodies [133].

It would be of high priority to learn whether possible crossreactivity also exists for anti-pp65 antibodies isolated from the SLE patients, especially the C-terminal reactive antibodies, which showed a higher incidence in SLE patients.

In addition, a HCMV gB expressing adenovirus induced antibodies against dsDNA and the U1-70 kDa spliceosome (U1-70 k) protein in immunized mice [134]. In humans, however, conflicting data are reported on U1-70 k autoantibodies in healthy HCMV positive persons. Serum from healthy persons screened within a vaccination study was tested for the presence of U1-70 k autoantibodies and an increase in frequency and quantity in HCMV positive persons was found [135]. The authors suggested that HCMV may play a role in inducing autoimmune disease in a subset of these individuals. In contrast, in a following study no indications for a higher frequency of SLE autoantibodies were found in sera from healthy volunteers vaccinated with the Towne strain [136]. This latter finding was confirmed by a study applying vaccination using soluble recombinant gB in combination with an adjuvant MF59 and gB expressed by a recombinant canarypox virus [137], leaving the question concerning the relation between gB antibodies and autoantibodies open.

### 4.3. HCMV and T1D

So far unknown mechanisms by which HCMV could interfere with immunity and cause T1D have been postulated. For instance, HCMV inclusion bodies were found in pancreatic island of children with fatal HCMV infection [138], suggesting that HCMV infection may cause immune reactions and destruction of these cells. However in other autopsy samples of pancreas tissue from T1D patients no indication for HCMV infection was found by nested PCR [139], even though the ability of HCMV to infect *β*-cells* in vitro* has been reported [140]. While HCMV- or immune-mediated cell death is a possible outcome of abortive HCMV infection, the data should be carefully interpreted since only IE1/pp72 and pp65 proteins were detected in these cultures. Firstly, pp65 protein is abundantly present in the HCMV particle and therefore viral* de novo* gene expression is not necessarily needed for detection of pp65 and, secondly, IE/pp72 expression is in most cases possible to detect after virus entry in various cells, also in nonpermissive cell types, but does not necessarily implicate successful HCMV replication. To prove this assumption, evidence of late gene expression or release of infectious viral progeny should be provided.

Moreover, mice vaccinated with the HCMV strain AD169 were found to produce antibodies against a 38 kDa human islet cell protein [141]. There is no further follow-up on this finding reported and the HCMV antigen possibly causing the crossreactive antibodies (the antigen should be a virion component since HCMV does not replicate in mouse cells) was not identified and it is also not clear with which host protein the antibodies may crossreact and whether the antibodies have indeed the potential to induce autoimmunity.

T-cells play a major role in destruction of pancreatic *β*-cells in T1D patients [142] and there is a strong correlation of T1D disease with certain alleles in the highly polymorphic MHC II locus [143]. It has been shown that GAD65 (glutamic acid decarboxylase) specific autoreactive CD4+ T-cells involved in T1D and stiff-man syndrome also recognize a HCMV UL57 derived peptide. The UL57 peptide can be processed and presented by dendritic cells, suggesting a potential involvement in loss of CD4+ T-cell tolerance to GAD65 [144].

## 5. T-Cells, HCMV, and RA

CD8+ T-cells are of particular importance to control HCMV replication. A hallmark of HCMV infection is the extraordinary expansion of HCMV specific CD8+ T-cells. It is becoming evident though that CD4+ T-cells might be even more important to prohibit HCMV associated disease [145] and that HCMV specific CD4+ T-cells are found in HCMV positive persons at a high frequency. These T-cells are typically end-differentiated effector memory cells, which have lost expression of CD27 and CD28, but express CD45RA and are therefore classified as effector memory or EMRA cells. HCMV specific CD4+ CD28− CD27− T-cells express granzyme B and have been shown to possess both cytotoxic activity and the ability to express IFN*γ* upon encountering of HCMV antigens [23, 146]. Similarly, expanded CD4+ T-cell populations are also documented in patients with various autoimmune diseases, including RA [147, 148], MS [149], and Wegener's granulomatosis [150]. However, there is growing evidence that the HCMV seropositive population of the patients is causing the association with disease and is responsible for the extraordinary expansion of CD4+ CD28− T-cells [100, 148, 151–153].

### 5.1. CD4+ T-Cells in HCMV Infected RA Patients

In RA patients T-cells are strongly implicated in disease pathogenesis. This is characterized by a large and stable clonal expansion of T-cells and increased frequencies (up to 10%) of CD4+ CD28− cells [154–156]. Expansion of the CD4+ CD28− subset was particularly pronounced in HCMV positive RA patients [100, 148, 152, 153], indicating that the disease* per se* is probably not the cause for the strong expansion of CD4+ CD28− cells, but the coincidence of disease and HCMV infection. Actually it was observed that the CD4+ CD28− T-cell population was about 3-fold higher in HCMV positive RA patients than in healthy HCMV positive subjects [100], suggesting that the disease contributes essentially to the expansion of CD4+ CD28− T-cells.

The majority of the CD4+ CD28− cells were found to be HCMV-specific [148] and no autoreactivity could be measured in that particular study, although CD4+ CD28− T-cells in RA patients were found to be autoreactive in a previous study [156] and the CD4+ CD28− cells were only partially susceptible to the control by regulatory T-cells. Interestingly, it was recently suggested that HCMV-specific CD4+ CD28− CD27− cells are able to function as regulatory T-cells downregulating both HCMV-specific and to a lesser extent unrelated immune responses. This function was shown to be dependent on granzyme B and to some extent on TGF*β* [157]. If indeed the HCMV specific CD4+ CD28− CD27− cells are by themselves, at least partly, able to confer regulatory T cell functions, additional downregulation might not be easy to detect.

The question remains whether the HCMV-specific CD4+ CD28− cells play a role for the course of the disease. Although no significant increase in HCMV seroprevalence was observed in RA patients, a significant association of HCMV IgG and progression of joint destruction and number of required surgical procedures could be determined [100]. It was put forward that the increased CD4+ CD28− T-cell population in these patients might be involved in aggravated joint disease, as expanded CD4+ CD28− T-cells have been found to cause a significantly faster progression of joint destruction [158]. Despite the fact that CD4+ CD28− T-cells are also associated with severe extra-articular disease [159], this association was not noted for HCMV seropositivity [160].

### 5.2. Role for CD8+ T-Cells in RA

Studies on CD8+ T-cells from inflamed lesion of RA patients revealed enrichment of EBV and HCMV specific CD8+ T-cells in comparison to cells from peripheral blood. Although the ratio of EBV and HCMV specific synovial lymphocytes to blood lymphocytes was conspicuously higher in RA patients, the presence of virus specific T-cells was also found in inflamed lesions of patients suffering from various autoimmune diseases (psoriatic arthritis, Reiter's syndrome, ankylosing spondylitis, and arthrosis) [161]. These findings prompted the authors of the study to propose that the virus specific CD8+ T-cells do not play a role for initiation of disease but rather are a consequence of preferential homing of the cells to inflamed tissue after virus reactivation. Furthermore, whereas the virus-specific CD8+ T-cells were readily observed in chronic stages, they were not found in samples drawn early after onset of disease, again arguing against a role in initiation of the disease process [161].

These HCMV specific CD8+ T-cells might contribute to RA-induced inflammation in the tissue by local release of proinflammatory cytokines. Recently, a possible TCR-independent proliferation of HCMV-specific CD8+ T-cells was proposed [162]. Particularly T-cells (CD8^+^ CD45RO^+^) specific for HCMV antigens presented by HLA-A*02:01, but not by HLA-B*07:01, were found to proliferate in an IL-15 dependent fashion [162]. This is interesting because high expression of IL-15 is found both in the synovial fluid and serum of RA patients and has been found to attract and activate T-cells, thereby contributing to the pathogenesis of RA [163]. It is therefore tempting to speculate that the high prevalence of HCMV-specific CD8+ T-cells in the joints of RA patients might be connected to IL-15 expression.

In light of this, it should not escape our attention that pp65 specific, HLA-C restricted CD8+ T-cells were found to crossreact with an HLA-DR4 alloantigen [164], demonstrating an additional level, at which loss of immune tolerance can occur in HCMV infection. Moreover, it might be a unique characteristic of CMV infections that CD8+ T-cells are activated against highly promiscuous epitopes presented by MHC class II molecules, as demonstrated in rhesus cytomegalovirus infection [165]. This might provide additional challenges for the immune system concerning the maintenance of immune tolerance. The existence of such MHC II-restricted CD8+ T-cell responses in humans remains to be demonstrated.

## Figures and Tables

**Table 1:** Immunomodulatory functions by HCMV.

Gene	Phenotype/suggested mechanism	Reference
T-cell responses		
US2	Degrades MHC class I and II	[30, 31]
US3	Retention of MHC class I complexes, mislocalization of MHC class II	[32, 33]
US6	Inhibits the peptide transporter TAP	[34, 35]
US11	Degrades MHC class I	[36]
US8	Binds MHC class I heavy chains, function unknown	[37]
UL18	Inhibits MHC I interaction with the PLC	[38]
miR-US4-1	Targets Erap1	[39]
?	Disturbed interaction between MHC class I and the PLC	[40]
UL11	Interacts with CD45	[41]

NK and NKT cell responses		
UL16	Intracellular retention of MICB and ULBP1 and 2	[42]
UL18	LIR-1 activation	[43]
UL40	Encodes a signal peptide for HLA-E	[44]
UL142	MICA and ULBP3 inhibition	[45, 46]
miR-UL112	MICB downregulation	[47]
UL83	Inhibition of NKp30	[48]
TRL11	Inhibition of CD16 activation	[49]
UL119-118; TRL11	Inhibition of CD16 activation	[49]
US10	Downregulates HLA-G	[50]
US2	Inhibits CD1d	[51]

Cytokines, chemokines, chemotaxis		
US28	Chemokine receptor; promotes chemotaxis; potential chemokine trap	[52, 53]
US27	Enhancement of CXCR4 signaling	[54]
UL33	GPCR family; modulates CCR5 and CXCR4	[55]
UL111a	vIL-10; hIL-10-like functions	[56, 57]
UL111a	LAvIL10; downregulates MHC class II expression	[58]
UL146	vCXCL1, hCXCR1/hCXCR2-specific chemokine; promotes neutrophil chemotaxis	[59]
UL147	IL-8 similarity	[60]
UL21.5	Sequesters RANTES; binds CCL5 and prevents host cell signalling	[61]
UL128	Increases TNF*α* and IL-6 expression by PBMC; promotes PBMC migration	[62]
UL78	GPCR family member; modulates CCR5 and CXCR4	[55]

Modulation of IFN and TNF signaling		
UL138	Upregulates TNF*α*R1	[63, 64]
UL144	TNFR homolog, inhhibits T-cell prolif. via BTLA-4; induces CCL22	[65]
UL7	Inhibits inflammatory cytokine production	[66]
?	Jak1 degradation	[67]
?	STAT2 degradation	[68]
UL83	Suppresses type I IFN response and IRF3-induction	[69]
TRS1	Blocks PKR-mediated translation shut-off	[70]
UL126a	Inhibits OAS	[71]
UL123	Binds STAT2 and inhibits DNA binding; modulated phosphorylation state of IE1	[72]
UL122	Inhibiton of NF*κ*B; inhibition of IFN*β* transcription	[73]

Apoptosis		
UL37x1	Neutralizes Bax	[74]
UL36x1	Caspase-8 inhibitor	[75]
UL121/123	Inhibits apoptosis	[76]

Intrinsic immunity		
UL123	Disruption of ND10	[77]
UL82	Daxx inhibitor	[78–80]

**Table 2:** Prevalence of HCMV specific IgG and IgM in autoimmune disease patients.

Nationality/ethnic background/group	Disease				Control group					Reference
	Nr^a^	Age^b^	HCMV seropositivity		Group	Nr	Age	HCMV seropositivity	
			% IgG	% IgM				% IgG	% IgM
Systemic sclerosis										
Turkey	46	45	96		Primary antiphospholipid syndrome; healthy	38; 65	36; 35	95; 95		[81]
Caucasians	137		66		Patients with osteoarthritis, fibromyalgia, gout or regional musculoskeletal pain syndromes	145		69		[82]
Swiss	86	56	59	20	RA; osteoarthritis	43; 43	56	12; 21	7; 16	[83]

Systemic lupus erythematosus										
Turkey	198	38	100		Primary antiphospholipid syndrome; healthy	38; 65	36; 35	95; 95		[81]
French/inactive SLE; active SLE	76; 42	34; 35	76; 83		Healthy	31	33	58		[84]
Taiwanese	87		97	10	Cerebral vascular accident patients	97		100	1	[85]
variable	36	15	42		Matched sibling/relative/friend	36	16	47		[86]
African American	144		81	11	Randomly selected from driver's license agencies	72	Matched	79	8	[87]
White	86		55	9	Randomly selected from driver's license agencies	204	Matched	57	5	[87]
variable	196	45	66		Healthy	392	46	69		[88]
Norwegian	20	54	95	45	Healthy	26	49	69	4	[89]
Italian	60	41	82	5	Blood donors	100	40	69	3	[90]
British	97		91		RA; healthy	50; 97		64; 43		[91]
Variable	117	<20	36		Healthy	153	Matched	26		[92]

Type 1 Diabetes Mellitus										
Finnish	169	1.3	23		Healthy, matched for *HLA-DQB1 *	791	1.3	26		[93]
Finnish	90	<7	47	4	Healthy	90	<7	42	4	[94]
Finnish	11	<2	18		Healthy siblings	11	<2	56		[94]

Multiple sclerosis										
Swedish	658	35	57		Healthy	786	36	65		[95]
Iranian	78	29	98		Healthy	123	29	52		[96]
Variable (USA)	189	15	28		Healthy	66	15	36		[97]
variable	144		50		Healthy	288		56		[1]
Norwegian	144	39	63		Traumatic fractures or rupture, gynaegological or plastic surgery disorder	170	40	69		[98]
Spanish	41	39	78		OND (other neurological disease	31	48	85		[99]

Rheumatoid Arthritis										
German	202		57		Blood bank	272	Matched	54		[100]
Swiss	43	56	12	7	Osteoarthritis	43	56	21	16	[83]
English	50		64		Healthy	97		43		[91]

Autoimmune thyroid disease										
Greece	34	10	50		Healthy	31	9	55		[101]

Sjögren's syndrome										
English	24		79		Healthy (dental reatment)	24	Matched	63		[102]

^a^ Number of subjects.

^b^ Median age of subjects.

## Final Remarks

At first glance a number of HCMV's characteristic attributes appear to predispose this particular virus to be more likely involved in the induction of autoimmune disorders than many other pathogens. Commonly found in all human populations, lasting throughout life in alternating cycles of productive and latent infection, establishing lytic replication in multiple tissues, expressing a plethora of antigens, manipulating extensively intrinsic, innate, and adaptive immune functions, HCMV infection should hazard many opportunities to induce a loss of immunological tolerance and cause autoimmune disease. On the other hand, the cospeciation of HCMV with its human host over millions of years has obviously led to an optimally adapted opportunist which does little harm and avoids clinical symptoms in the immunocompetent host. Evidently, anti-inflammatory strategies of HCMV [57, 166] can effectively counterbalance immune activation and tissue damage.

We have surveyed the currently available literature for evidence on HCMV association with onset or exacerbation of certain autoimmune disease. While a causative linkage between HCMV and systemic lupus erythematosus, systemic sclerosis, diabetes mellitus type 1, and rheumatoid arthritis is suggested by some findings reported by the literature, the overall evidence does not convincingly support this notion. Albeit an association of HCMV seroprevalence and AID could not be established, HCMV could still play a coresponsible role for the onset of disease, disease progression, or amelioration. For convincing conclusions population-based prospective studies are urgently needed in the future.

Several studies provide evidence for an exciting interplay between adaptive immune responses to HCMV and manifestations of certain autoimmune disorders, for example, in RA and SLE. Specifically, immunopathogenic mechanisms by which HCMV could contribute to the course of autoimmune disease have been indicated, for example, molecular mimicry by the UL94 antigen in SSc patients and UL83/pp65 in SLE patients, as well as aggravation of joint inflammation by induction and expansion of CD4+/CD28− T-cells in HCMV infected RA patients. Future studies should provide more detailed insight into the immunopathological potential of HCMV-reactive immune cells to develop potential new strategies of targeted therapeutic intervention.
